# Supplementary material for: Vibrational enhancement of quadrature squeezing and phase sensitivity in resonance fluorescence
Source: Nat Commun. 2019 Jul 10;10:3034. doi: 10.1038/s41467-019-10909-3 (PMC6620290; doi:10.1038/s41467-019-10909-3)
Supplement: Supplementary file 1 — Supplementary Information [file 41467_2019_10909_MOESM1_ESM.pdf]

SUPPLEMENTARY INFORMATION
VIBRATIONAL ENHANCEMENT OF QUADRATURE SQUEEZING AND PHASE SENSITIVITY IN RESONANCE FLUORESCENCE

Jake Iles-Smith, et al

SUPPLEMENTARY NOTE 1 - VARIATIONAL POLARON TRANSFORMATION

The starting point for our quantum dot model is a Hamiltonian describing a two level emitter (TLE) with ground and excited states $|g\rangle$ and $|e\rangle$ respectively, driven by a classical laser field with frequency $\omega_{\rm l}$ and Rabi frequency Ω. As introduced in the main text we have,

$$H = \hbar\delta\sigma^\dagger\sigma + \frac{\hbar\Omega}{2}\sigma_{\rm x} + \hbar\sigma^\dagger\sigma\sum_k g_k(b_k^\dagger + b_k) + \hbar\sum_m \left(h_m\sigma^\dagger a_m e^{i\omega_{\rm l}t} + \text{h.c.}\right) + \sum_k \hbar\omega_k b_k^\dagger b_k + \sum_m \hbar\nu_m a_m^\dagger a_m, \tag{1}$$

where $\sigma = |g\rangle\langle e|$ and $\sigma_{\rm x} = \sigma + \sigma^\dagger$. The Hamiltonian is written in a frame rotating with respect to the laser frequency $\omega_{\rm l}$ and in the rotating wave approximation, with the laser and QD transition detuned by $\delta = \omega_0 - \omega_{\rm l}$. To model the dynamics of the TLE, we apply the variational polaron transformation to the above Hamiltonian, allowing us to derive a master equation valid outside of the weak electron-phonon coupling regime [1–3]. The variational polaron transformation is given by the state dependent displacement operator

$$\mathcal{U}_{\rm V} = |g\rangle\langle g| + |e\rangle\langle e|B_+, \tag{2}$$

where $B_\pm = \exp\left[\pm\sum_k f_k(b_k^\dagger - b_k)/\omega_k\right]$ is a multi-mode displacement operator, and f_k is a parameter which we shall use to variationally optimise the transformation in the next section.

Applying this transformation to the initial Hamiltonian yields $H_{\rm V} = \mathcal{U}_{\rm V} H \mathcal{U}_{\rm V}^\dagger = H_{\rm r} + H_{\rm I}^{\rm em} + H_{\rm I}^{\rm ph} + H_{\rm B}$, where $H_{\rm r} = \hbar\delta_{\rm r}\sigma^\dagger\sigma + (\hbar\Omega_{\rm r}/2)\sigma_{\rm x}$, and $H_{\rm B} = H_{\rm B}^{\rm em} + H_{\rm B}^{\rm ph}$, with $H_{\rm B}^{\rm ph} = \hbar\sum_k \omega_k b_k^\dagger b_k$ and $H_{\rm B}^{\rm em} = \hbar\sum_m \nu_m a_m^\dagger a_m$. Notice that we have introduced the renormalised detuning $\delta_{\rm r} = \delta + R$, with $R = \sum_k \omega_k^{-1} f_k(f_k - 2g_k)$, and renormalised driving strength $\Omega_{\rm r} = \Omega B$, where $B = \langle B_\pm\rangle_{H_{\rm B}} = \text{tr}_{\rm B}(B_\pm\rho_{\rm B}^{\rm ph})$ is the expectation value of the displacement operator with respect to the thermal state $\rho_{\rm B}^{\rm ph} = \exp\left(-\beta H_{\rm B}^{\rm ph}\right)/\,\text{tr}_{\rm B}\left[\exp(-\beta H_{\rm B}^{\rm ph})\right]$ with $\beta = 1/k_{\rm B}T$ the inverse temperature. The transformed interaction Hamiltonians read

$$H_{\rm I}^{\rm ph} = \frac{\hbar\Omega}{2}\left(\sigma_{\rm x}B_{\rm x} + \sigma_{\rm y}B_{\rm y}\right) + \hbar\sigma^\dagger\sigma B_{\rm z} \quad\text{and}\quad H_{\rm I}^{\rm em} = \hbar\sum_m h_m\sigma^\dagger B_+ a_m e^{i\omega_{\rm l}t} + \text{h.c.} \tag{3}$$

where $B_{\rm x} = (B_+ + B_- - 2B)/2$, $B_{\rm y} = i(B_+ - B_-)/2$, and $B_{\rm z} = \sum_k (g_k - f_k)(b_k^\dagger + b_k)$. Notice that the electron–phonon interaction now contains a mixture of displacement operators and linear coupling. In the next section we outline how the variational principle can be used to specify the contribution of each of these terms.

Minimising the free energy

As mentioned above, we now choose the displacement such that the transformed Hamiltonian minimises the free energy of the system. We do so by minimising the Feynman–Bogliubov upper bound on the free energy [3], that is:

$$A_{\rm B} = -\frac{1}{\beta}\ln\left[\text{tr}(e^{-\beta H_0})\right] + \langle H_{\rm I}^{\rm ph}\rangle_{H_0} + \mathcal{O}\left(\langle H_{\rm I}^2\rangle_{H_0}\right),$$

where $H_0 = H_{\rm r} + H_{\rm B}$. This procedure allows us to derive a master equation which is valid over a broad range of parameters, and does not suffer from the same pathologies as standard polaron theory (see Refs. [2] and [3] for a detailed discussion). Thus, minimising $A_{\rm B}$ with respect to f_k, we obtain the expression

$$\frac{\partial A_{\rm B}}{\partial f_k} = \frac{1}{\text{tr}\left[e^{-\beta H_0}\right]}\,\text{tr}\left[\frac{\partial H_0}{\partial f_k}e^{-\beta H_0}\right] = 0. \tag{4}$$

Solving this equation, and substituting the minimised displacements into the expressions for the renormalised system parameters, we find that in the continuum limit we have:

$$\Omega_{\rm r} = \Omega B = \Omega\exp\left[-\frac{1}{2}\int_0^\infty \frac{J(\omega)F^2(\omega)}{\omega^2}\coth\left(\frac{\hbar\beta\omega}{2}\right)d\omega\right] \quad\text{and}\quad \delta_{\rm r} = \delta + \int_0^\infty \frac{J(\omega)F(\omega)}{\omega}(2 - F(\omega))d\omega, \tag{5}$$

where we have introduced the phonon spectral density $J(\omega) = \sum_k |g_k|^2\delta(\omega - \omega_k)$ and the variational function:

$$F(\omega) = \frac{\eta_{\rm r} - \delta_{\rm r}\tanh\left(\hbar\beta\eta_{\rm r}/2\right)}{\eta_{\rm r} - \tanh\left(\hbar\beta\eta_{\rm r}/2\right)\left(\delta_{\rm r} - \frac{\Omega_{\rm r}^2}{2\omega}\coth\left(\hbar\beta\omega/2\right)\right)}. \tag{6}$$

Here $\eta_{\rm r} = \sqrt{\delta_{\rm r}^2 + \Omega_{\rm r}^2}$ is the renormalised generalised Rabi frequency. These equations can be solved self-consistently to find the renormalised parameters that minimise the Feynman–Bogliubov free energy.

SUPPLEMENTARY NOTE 2 - VARIATIONAL MASTER EQUATION

To describe the dynamics of the reduced state of the TLE, $\rho_{\rm V}(t)$, we shall treat the interaction Hamiltonian $H_{\rm I} = H_{\rm I}^{\rm em} + H_{\rm I}^{\rm ph}$ to second order using a Born-Markov master equation in the variational polaron frame, which in the interaction picture takes the form [4]:

$$\frac{\partial \tilde{\rho}_{\rm V}(t)}{\partial t} = -\frac{1}{\hbar^2}\int_0^\infty d\tau \;\; {\rm tr_B}\left[H_{\rm I}(t), \left[H_{\rm I}(t-\tau), \tilde{\rho}_{\rm V}(t)\otimes\rho_{\rm B}^{\rm em}\otimes\rho_{\rm B}^{\rm ph}\right]\right], \tag{7}$$

where $\tilde{\rho}_{\rm V}(t) = \exp[iH_0 t]\rho_{\rm V}(t)\exp[-iH_0 t]$ and $H_{\rm I}(t) = \exp[iH_0 t]H_{\rm I}\exp[-iH_0 t]$ are interaction picture operators, and we have made the Born approximation which is to factorise the environmental density operators, here in the variational polaron frame, such that they remain static throughout the evolution of the system. Note that correlations may be generated between the system and the phonon environment in the original representation. We shall assume that in the variational polaron frame the phonon environment remains in the thermal state defined above, while the electromagnetic environment remains in its vacuum state $\rho_{\rm B}^{\rm em} = \bigotimes_m |0_m\rangle\langle 0_m|$. Since the trace over the chosen states of the environments removes terms linear in creation and annihilation operators, we may split the master equation into two separate contributions corresponding to the phonon and photon baths respectively [5],

$$\frac{\partial \tilde{\rho}_{\rm V}(t)}{\partial t} = \mathcal{K}_{\rm ph}[\tilde{\rho}_{\rm V}(t)] + \mathcal{K}_{\rm em}[\tilde{\rho}_{\rm V}(t)]. \tag{8}$$

In the subsequent sections we shall analyse each of these contributions in turn.

Phonon contribution

To derive the contribution from the phonon environment, we follow Ref. [1]. We start by transforming into the interaction picture with respect to the Hamiltonian $H_0 = \frac{\hbar\delta_{\rm r}}{2}\sigma_{\rm z} + \frac{\hbar\Omega_{\rm r}}{2}\sigma_{\rm x} + \frac{\hbar R}{2}\mathbb{1} + \hbar\sum_k \omega_k b_k^\dagger b_k + \hbar\sum_m \nu_m a_m^\dagger a_m$. Using this transformation, the phonon component of the interaction Hamiltonian takes the form

$$H_{\rm I}^{\rm ph}(t) = \frac{\hbar\Omega}{2}\left(\sigma_{\rm x}(t)B_{\rm x}(t) + \sigma_{\rm y}(t)B_{\rm y}(t)\right) + \hbar\sigma^\dagger\sigma(t)B_{\rm z}(t). \tag{9}$$

Here $B_{\rm x}(t) = \frac{1}{2}(B_+(t) + B_-(t) - 2B)$, $B_{\rm y}(t) = \frac{i}{2}(B_+(t) - B_-(t))$, and $B_{\rm z}(t) = \sum_k (g_k - f_k)(b_k e^{-i\omega_k t} + b_k^\dagger e^{i\omega_k t})$, where $B_\pm(t) = \exp[\pm\sum_k \frac{f_k}{\omega_k}(b_k^\dagger e^{i\omega_k t} - b_k e^{-i\omega_k t})]$. The system operators can be formally written in the interaction picture as $\sigma_\alpha(t) = \sum_{jk}\sigma_\alpha^{jk}e^{i\lambda_{jk}t}|\psi_j\rangle\langle\psi_k|$, where $|\psi_j\rangle$ are the eigenstates of the renormalised system Hamiltonian satisfying $H_{\rm r}|\psi_j\rangle = \psi_j|\psi_j\rangle$, $\hbar\lambda_{jk} = \psi_j - \psi_k$, and $\sigma_\alpha^{ij} = \langle\psi_i|\sigma_\alpha|\psi_j\rangle$ with $\alpha \in \{{\rm x},{\rm y},{\rm z}\}$. Using these expressions and moving back into the Schrödinger picture we arrive at the form of $K_{\rm ph}$ given in the main text.

Photon contribution

We now focus on the interaction between the electromagnetic field and the TLE. The interaction picture Hamiltonian for the field may be written as $H_{\rm I}^{\rm em}(t) = \sigma^\dagger(t)e^{i\omega_{\rm l}t}B_+(t)A(t) + {\rm h.c.}$, where $A(t) = \hbar\sum_m h_m a_m e^{-i\omega_m t}$ and $B_+(t)$ is as given in the previous section. If we consider the interaction picture transformation for the system operators we have

$$\sigma(t){\rm e}^{-i\omega_{\rm l}t} = \exp\left[i\left(\frac{\delta_{\rm r}}{2}\sigma_{\rm z} + \frac{\Omega_{\rm r}}{2}\sigma_{\rm x}\right)t\right]\sigma\exp\left[-i\left(\frac{\delta_{\rm r}}{2}\sigma_{\rm z} + \frac{\Omega_{\rm r}}{2}\sigma_{\rm x}\right)t\right]{\rm e}^{-i\omega_{\rm l}t} \approx \sigma e^{-i\omega_0 t}, \tag{10}$$

where we have used the fact that $\omega_{\rm l} \gg \Omega_{\rm r},\ \delta_{\rm r}$ for typical solid-state emitters to simplify the interaction picture transformation [1, 6]. By substituting this expression into the photon contribution of the master equation and

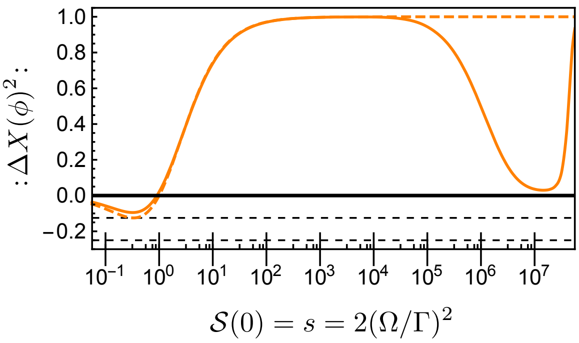

Supplementary Figure 1. Quadrature variance for resonant excitation $\delta = 0$, plotted as a function of driving strength from well below to well above saturation. The phonon enhanced coherent scattering regime can be seen as a decrease in the quadrature variance around $\mathrm{S}(0) \approx 10^5$, though this does not giving rise to quadrature squeezing as the normally ordered variance remains positive.

recalling that all modes of the field are in their vacuum state, we have

$$\mathcal{K}_{\mathrm{em}}[\tilde{\rho}_{\mathrm{V}}(t)] = -\frac{1}{\hbar^2}\int_0^\infty d\tau\ \mathrm{tr}_{\mathrm{B}}\left[H_{\mathrm{I}}^{\mathrm{em}}(t), [H_{\mathrm{I}}^{\mathrm{em}}(t-\tau), \tilde{\rho}_{\mathrm{V}}(t) \otimes \rho_{\mathrm{B}}^{\mathrm{em}}]\right] = \gamma(\omega_0)\left(\sigma\tilde{\rho}_{\mathrm{V}}(t)\sigma^\dagger - (1/2)\left\{\sigma^\dagger\sigma, \tilde{\rho}_{\mathrm{V}}(t)\right\}\right), \tag{11}$$

with the anticommutator $\{A, B\} = AB + BA$, and where the spontaneous emission rate is given by [1, 7, 8]

$$\gamma(\omega_0) = \mathrm{Re}\left[\int_0^\infty e^{i\omega_0\tau} G(\tau)\Lambda(\tau) d\tau\right]. \tag{12}$$

Here $\Lambda(\tau) = \int_0^\infty d\nu J_{\mathrm{em}}(\nu) e^{i\nu\tau}$, with $J_{\mathrm{em}}(\nu) = \sum_m |f_m|^2 \delta(\nu - \nu_m)$ being the spectral density of the electromagnetic environment, and $G(\tau)$ is a phonon correlation function given by

$$G(\tau) = B^2 \exp\Big[\int_0^\infty \frac{J(\omega)F(\omega)^2}{\omega^2}\Big(\coth(\hbar\beta\omega/2)\cos\omega\tau + i\sin\omega t\Big) d\nu\Big]. \tag{13}$$

As discussed in the manuscript, the local density of states of the electromagnetic field does not vary appreciably over energy scales relevant to QD systems in bulk, which allows us to make the assumption that the spectral density is flat [1, 6], $J_{\mathrm{em}}(\nu) \approx 2\Gamma/\pi$. The electromagnetic correlation function may then be evaluated as $\Lambda(\tau) \approx \Gamma\delta(\nu) + i\mathcal{P}[1/\tau]$, where $\mathcal{P}$ denotes the principal value integral. Combining these expressions and resolving the remaining integral, we find that the spontaneous emission rate takes on the form $\gamma(\omega_0) \approx \Gamma$, where we have used the fact that $G(0) = 1$, such that in the Schrödinger picture we have

$$\mathcal{K}_{\mathrm{em}}[\rho_{\mathrm{V}}(t)] = \Gamma\mathcal{L}_\sigma\left[\rho_{\mathrm{V}}(t)\right] = \Gamma\left(\sigma\rho_{\mathrm{V}}(t)\sigma^\dagger - (1/2)\left\{\sigma^\dagger\sigma, \rho_{\mathrm{V}}(t)\right\}\right). \tag{14}$$

Although for a flat optical spectral density the variational polaron transformation does not alter the spontaneous emission rate, it does directly influence the first-order correlation function, and therefore the emission spectrum of the system. As discussed in detail in Refs. [9, 10], when calculated in the variational polaron frame, the steady-state first-order correlation function takes the form,

$$g^{(1)}(\tau) = G(\tau)\langle\sigma^\dagger(\tau)\sigma\rangle_{\mathrm{V}}, \tag{15}$$

where the first factor is the phonon correlation function, which leads to the emergence of a non-Markovian phonon sideband in the emission spectrum [7–10]. The second term is the first-order correlation function calculated in the variational polaron frame, and describes pure optical emission processes from the TLE. The phonon sideband contribution is particularly important for the coherent scattered power, which is given by the long-time limit of Eq. (15). That is, we have $P_{\mathrm{coh}} = \lim_{\tau\to\infty} g^{(1)}(\tau) = |\langle\sigma\rangle|^2 = B^2|\langle\sigma\rangle_{\mathrm{V}}|^2$, with the prefactor B^2 acting to decrease the overall fraction of coherently scattered light [9].

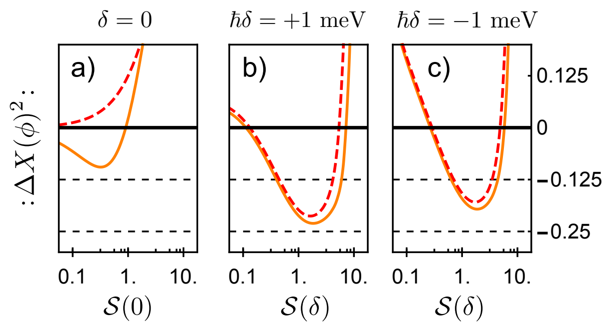

Supplementary Figure 2. Normalised quadrature variance calculated using our full phonon model without (orange, solid curves), and with (red, dashed curves) additional non-phonon induced pure-dephasing. This dephasing results in no squeezing below saturation on resonance, but the squeezing in the phonon-enhanced regime remains.

SUPPLEMENTARY NOTE 3 - QUADRATURE VARIANCE IN THE RESONANT PHONON-ENHANCED SCATTERING REGIME

As explained in the main text, quadrature squeezing in resonance fluorescence occurs when the total power P and the coherently scattered power P_{coh} satisfy the condition $:\Delta X(\phi)^2 := 1 - |2P-1| - 4P_{\text{coh}} < 0$. As such, it makes sense to first explore the regime of phonon-enhanced coherent scattering identified in Ref. [1], in which P_{coh} was shown to take on significant values in the strong driving regime on resonance. As can be seen in Supplementary Figure 1, however, on resonance, no squeezing occurs above saturation, although a reduction in the quadrature variance is apparent. This is because in this regime the coherent and incoherent contributions become approximately equal, and we have $P = 0.5$ and $P_{\text{coh}} = 0.25$, resulting in $:\Delta X(\phi)^2 :\approx 0$.

SUPPLEMENTARY NOTE 4 - ADDITIONAL DEPHASING EFFECTS

Our model described above includes spontaneous emission processes and effects caused by coupling to longitudinal acoustic phonons, which can be of both dephasing and dissipation in nature. The phonon enhanced coherent scattering and associated squeezing processes described in the main text take place in a regime where the phonon effects included in the phonon dissipator K_{ph} dominate. As such, the emergence of the phonon enhanced regime is robust against other (non-phonon induced) dephasing processes that are weak or moderate compared to the spontaneous emission rate. These additional dephasing processes, may be caused, for example, by charge fluctuations.

To demonstrate the robust nature of the phonon enhanced squeezing regime, in Supplementary Figure (2) we show a version of Figure (2) of the main text, which plots the normally ordered quadrature variance as a function of driving strength. The solid orange line is our theory including phonons and spontaneous emission but without additional pure-dephasing (identical to the solid orange curve in the main text), and the dashed red curve shows the effect of also including pure-dephasing, which we achieve by adding a term $\gamma(\sigma^\dagger\sigma\rho_{\text{V}}\sigma^\dagger\sigma - (1/2)\{\sigma^\dagger\sigma, \rho_{\text{V}}\})$ to the Schrödinger picture master equation, with the dephasing rate γ here equal to the spontaneous emission rate Γ. Interestingly, we see that this amount of pure-dephasing is sufficient to completely eliminate the squeezing below saturation that occurs in the absence of phonons, but the squeezing remains in the above saturation regime, with a magnitude nearly equal to that in its absence.

SUPPLEMENTARY NOTE 5 - EMITTED FIELD WIGNER FUNCTIONS

In order to elucidate the nature of the squeezed states of light produced, we can consider the emitted field Wigner function defined as $\mathcal{W}(x,p) = \pi^{-1}\int_{-\infty}^{\infty}\langle x+y|\rho_{\text{em}}|x-y\rangle \exp[-2ipy]dy$, where ρ_{em} is the state of the field. Following Ref. [11] we use the correspondence between the field operators and QD operators to associate the QD excited state $|e\rangle$ with the first field Fock state $|1\rangle$ and the QD ground state $|g\rangle$ with the field vacuum $|0\rangle$, such that $\rho_{\text{em}} = \sum_{n,m=0,1}(\rho_{\text{V}})_{nm}|n\rangle\langle m|$. In doing so, we expect $\mathcal{W}(x,p)$ to provide a qualitative representation of the electromagnetic field Wigner function for a class of measurements [12].

In Supplementary Figure 3 we show Wigner functions for the vacuum a), the squeezed state generated in the weak resonant excitation regime below saturation b), and the squeezed states generated only in the presence of

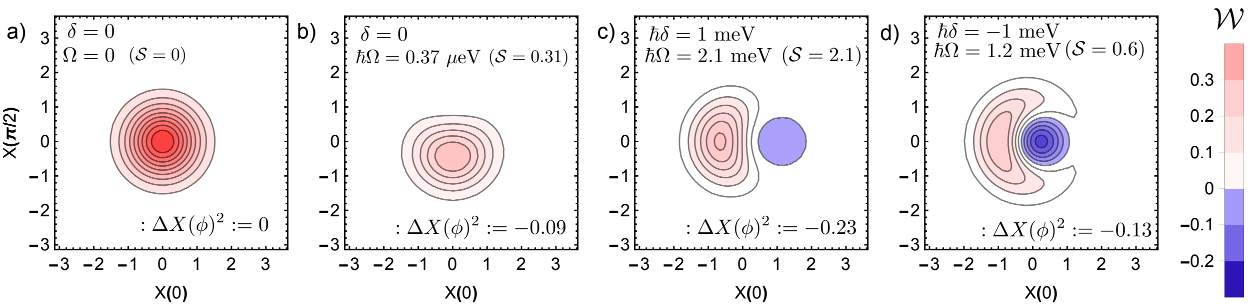

Supplementary Figure 3. Wigner functions for the vacuum state a), and three squeezed states generated in resonance fluorescence; on resonance and with weak driving b), and off resonant with strong driving c) and d). In cases c) and d) the squeezing occurs due to thermalisation, and can attain levels greater than in case b). Parameters as in all figures in the main text.

phonons above saturation for off-resonant driving c) and d). Interestingly, in the resonant case b), although the state generated is strictly speaking non-Gaussian, its Wigner function is nevertheless positive everywhere, and is not significantly dissimilar from a truly Gaussian displaced squeezed vacuum state [13]. However, in cases c) and d) the non-Gaussian nature of the field is quite apparent, with the Wigner functions taking on substantial negative values associated with non-classicality. Thus, in this regime a highly non-classical, quadrature squeezed and antibunched state is produced.

[1] McCutcheon, D. P. S. & Nazir, A. Model of the Optical Emission of a Driven Semiconductor Quantum Dot: Phonon-Enhanced Coherent Scattering and Off-Resonant Sideband Narrowing. *Phys. Rev. Lett.* **110**, 217401–217405 (2013).

[2] McCutcheon, D. P. S., Dattani, N. S., Gauger, E. M., Lovett, B. W. & Nazir, A. A general approach to quantum dynamics using a variational master equation: Application to phonon-damped rabi rotations in quantum dots. *Phys. Rev. B* **84**, 081305–081308 (2011).

[3] Nazir, A. & McCutcheon, D. P. S. Modelling exciton–phonon interactions in optically driven quantum dots. *J. Phys. Condens. Matter* **28**, 103002–103027 (2016).

[4] Breuer, H. P. & Petruccione, F. *The Theory of Open Quantum Systems* (Oxford University Press, Oxford, 2002).

[5] McCutcheon, D. P. S. Optical signatures of non-Markovian behavior in open quantum systems. *Phys. Rev. A* **93**, 022119–022125 (2016).

[6] Carmichael, H. *Statistical Methods in Quantum Optics 1: Master Equations and Fokker-Planck Equations* (Springer, 1998).

[7] Roy-Choudhury, K. & Hughes, S. Spontaneous emission from a quantum dot in a structured photonic reservoir: phonon-mediated breakdown of fermi's golden rule. *Optica* **2**, 434–437 (2015).

[8] Roy-Choudhury, K. & Hughes, S. Quantum theory of the emission spectrum from quantum dots coupled to structured photonic reservoirs and acoustic phonons. *Phys. Rev. B* **92**, 205406–205427 (2015).

[9] Iles-Smith, J., McCutcheon, D. P. S., Mørk, J. & Nazir, A. Limits to coherent scattering and photon coalescence from solid-state quantum emitters. *Phys Rev B* **95**, 201305–201310 (2017).

[10] Iles-smith, J., McCutcheon, D. P. S., Nazir, A. & Mørk, J. Phonon scattering inhibits simultaneous near-unity efficiency and indistinguishability in semiconductor single-photon sources. *Nat. Photonics* **11**, 521–527 (2017).

[11] Schulte, C. H. H. *et al.* Quadrature squeezed photons from a two-level system. *Nature* **525**, 222–225 (2015).

[12] Quijandría, F., Strandberg, I. & Johansson, G. Steady-state generation of wigner-negative states in one-dimensional resonance fluorescence. *Phys. Rev. Lett.* **121**, 263603–263608 (2018).

[13] Walls, D. & Milburn, G. *Quantum Optics* (Springer, 2008).
